# Finite Element Simulation and Experiment for Electromagnetic Flanging Forming of Aluminum Alloy Sheet

**DOI:** 10.3390/ma18184345

**Published:** 2025-09-17

**Authors:** Zhengrong Zhang, Jingchao Yao, Fei Wu, Jun Zhang, Chaojun Chen, Chun Huang

**Affiliations:** School of Material and Energy, Guangdong University of Technology, Guangzhou 510006, China; zzr@gdut.edu.cn (Z.Z.); 16650393359@163.com (J.Y.); zj2669178621@163.com (J.Z.); chenchaojunmx@outlook.com (C.C.);

**Keywords:** electromagnetic flanging, step coil, electromagnetic force distribution, numerical simulation

## Abstract

In order to address the problem of the large gap in the film on the straight edge of the electromagnetic flanging forming by the flat coil affecting the quality of the flanging part, a multi-layer variable-turn stepped coil is proposed. Numerical simulation analysis and experimental research were conducted on the electromagnetic flanging forming process of flat coil and stepped coil. Research shows that in the early stage of forming, the electromagnetic force of the flat coil is uniformly distributed at the edge of the hole and the middle of the deformation zone of the sheet metal, causing the upper surface of the middle of the deformation zone of the sheet metal to present radial compressive stress and tangential compressive stress, and the upper surface of the sheet metal at the fillet of the die to present radial tensile strain, tangential compressive strain and thickness direction compressive strain. The electromagnetic force of the step coil is mainly concentrated at the hole edge of the sheet metal, causing the upper surface in the middle of the deformation zone of the sheet metal to present radial tensile stress and tangential tensile stress, as well as radial tensile strain, tangential and thickness direction compressive strain. Under the flat coil, the sheet material mainly undergoes plastic deformation under the action of axial electromagnetic force and can only be bent into a curved edge. Under the stepped coil, the sheet metal undergoes plastic deformation simultaneously under the combined action of axial and radial electromagnetic forces and can be flipped into a vertical edge. The feasibility of the electromagnetic flanging forming of the stepped coil was verified through experiments, and the experimental results were basically consistent with the simulation results.

## 1. Introduction

With the development of multiple fields such as aviation, aerospace, and automobiles towards environmental protection and energy conservation, the lightweighting of components is one of the most effective measures for energy conservation and emission reduction. As a result, high-strength and lightweight alloy materials such as aluminum alloys have gained favor [1,2,3]. Flanging is a stamping method in which the outer edge or hole edge of a blank or semi-finished product is flanged along a certain curve to form a vertical edge. It is usually used to form complex workpieces, such as the flanging of the middle wall panels of passenger cars in locomotives and vehicles, the flanging of the foot door pressing iron of passenger cars, the flanging of the outer door panels of automobiles, the flanging of fuel tanks in motorcycles, and the flanging of small threaded holes in metal plates, etc. However, due to the relatively low forming performance of aluminum alloys at room temperature, it is difficult to flange large-sized metal plates by traditional stamping forming. The workpiece is prone to cracking and tearing along the circumference at the flange, and the springback is very serious, which seriously affects the forming quality.

Electromagnetic forming is a forming process that uses the strong pulsed magnetic field force generated by an instantaneous pulsed magnetic field on metal workpieces for plastic processing [4,5]. Due to the extremely high speed and strain rate of electromagnetic flanging forming, which can increase the forming limit of materials and effectively solve the problems faced by traditional flanging, scholars at home and abroad have invested a lot of research in the electromagnetic flanging forming process [6,7], and aluminum alloy has excellent magnetic conductivity, which makes it widely used in the field of electromagnetic forming. Zhang Lei [8], in research on the electromagnetic flanging of sheet materials, utilized the method of experiments combined with simulations. The results showed that increasing the discharge voltage and improving the coil structure could make the magnetic field intensity greater and the magnetic field distribution more uniform. Zhang Hang [9], in the preparation and research of FeSiAl SMC, proposed that the higher the magnetic permeability of the magnetic material, the stronger the magnetic induction intensity generated under the same external magnetic field. Ziqin Yan et al. [10] conducted electromagnetic flanging experiments on several groups of A5052 aluminum alloy plates with different prefabricated hole sizes using electromagnetic coils. The experiments showed that the radial electromagnetic force promoted the material flow in the flanging area and increased the flanging height. Lei Zhang et al. [11] conducted traditional flanging and electromagnetic flanging on 5052 aluminum alloy. The comparison results showed that compared with traditional flanging, electromagnetic flanging could achieve a greater flanging height. Meanwhile, as the discharge point voltage increased, the adaptability of the flanging part was improved.

At present, scholars at home and abroad almost all conduct research on the electromagnetic flanging forming process using flat coils, and few studies focus on the electromagnetic flanging forming process of stepped coils. When the flanging height reaches a certain level, the flanged part obtained by the electromagnetic flanging forming of the flat spiral coil is bent, and the middle part of the deformation zone fails to adhere well to the mold, resulting in low forming accuracy and poor flanging quality [8,12]. Therefore, this paper proposes a stepped coil to increase the flanging height while ensuring the flanging quality. The spatio-temporal distribution of the electromagnetic force of the two types of coils was studied, and a reasonable structure of the stepped flanged coil was analyzed and obtained.

## 2. Electromagnetic Flanging Finite Element Simulation

### 2.1. Electromagnetic Flanging Process Model

**Figure 1:**
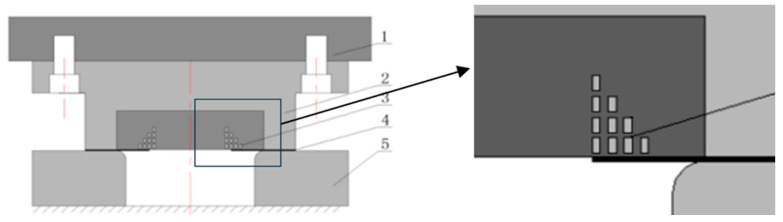
Geometry model of the process and three enlarged section views. 1-the blank holder fixed plate, 2-the blank holder, 3-the stepped coil, 4-the sheet metal with prefabricated hole, 5-the die.

The model of the electromagnetic flanging process for stepped coil round holes is shown in Figure 1, which includes the fixed plate of the flanging ring, the flanging ring, the stepped coil, the sheet material with prefabricated holes and the die. The inner diameter and fillet radius of the die are 110 mm and 8 mm, respectively. The model of the electromagnetic flanging process for the round hole of the flat coil is the same as that in Figure 1, except that the stepped coil is replaced with a flat coil. All coils are wound with flat copper wire, with a cross-sectional size of 2 × 4 mm. The inner diameter of the single-layer 4-turn flat coil is 70 mm, the outer diameter is 84 mm, and the turn spacing is 2 mm. When the number of turns and layers of the stepwise coil remains unchanged, increasing the relative distance d between the innermost copper wire of the coil and the edge of the sheet metal hole can enhance the concentration of the electromagnetic force of the sheet metal at the edge of the hole, making it easier to obtain a flanged part with better flanging quality. Therefore, the set 4-layer 4-turn stepped coil has the number of turns in each layer gradually decreasing from 4 turns to 1 turn from bottom to top, presenting a stepped distribution. The inner diameter of each coil is 60 mm, the outer diameter of the lowest layer is 74 mm, and the layer spacing is 1 mm. The turn spacing of each layer is the same as that of the flat coil. Compared with flat coils, although the manufacturing difficulty of stepped coils is greatly increased, the sheet metal speed response at the edge of the hole of stepped coils is faster. When the sheet metal in the deformation area is about to be attached to the mold, the sheet metal speed at the edge of the hole is smaller, which is more conducive to turning into a vertical edge.

### 2.2. Finite Element Model of Electromagnetic Field

**Figure 2:**
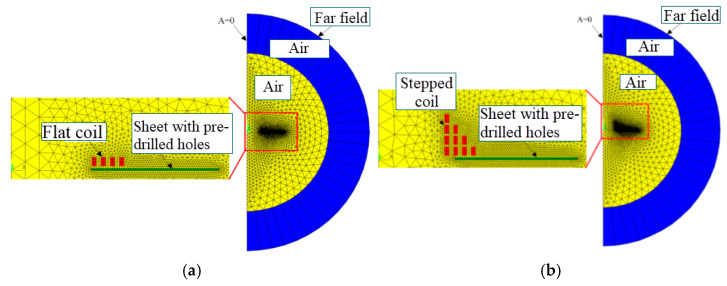
Finite element model for electromagnetic field analysis. (**a**) The flat coil; (**b**) the stepped coil.

As it is an axial symmetry problem, in order to improve the operational efficiency of the numerical simulation, only a finite element model of the electromagnetic flanging of a 1/2 circular hole needs to be established [13], as shown in Figure 2a,b, including four parts: the sheet with pre-drilled holes, the coil, the air, and the far field. The sheet metal with prefabricated holes and the coil are divided into mapping grids using the quadrilateral element Plane13 in the ANSYS APDL 2020 R2. The far field is divided into quadrilateral elements Inf110 by using the mapping grid technology. Considering that the air grid is affected by the deformation of the sheet metal and needs to be updated [14], it is divided into the triangular element Plane13 by using the free grid technology.

The material parameters of the electromagnetic field model are shown in Table 1.

**Figure 3:**
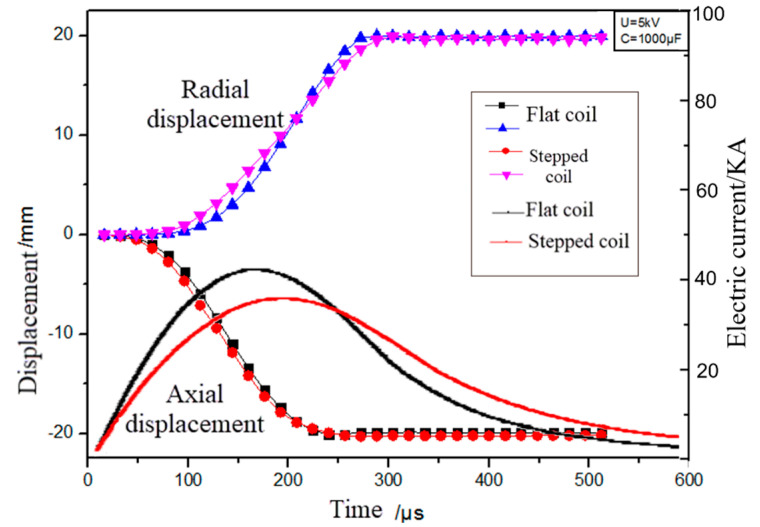
Discharge current curve and variation curve of displacement with time at hole edge of the sheet.

During the discharge process, the current data in the actual discharge circuit measured by the Rogowine coil and the oscilloscope were obtained, and the current waveform of the 1/2 cycle was fitted as shown, and the relationship between the sheet metal displacement at the hole edge and time is shown in Figure 3. Numerous studies at home and abroad have shown that the current after the first half of the wave in the discharge circuit has a very small influence on the electromagnetic forming process [15,16,17]. At the falling edge of the first half of the wave, the magnetic field intensity begins to decrease, and the distance between the sheet material and the coil is too large, causing the electromagnetic force acting on the sheet material to attenuate rapidly [18,19]. During the numerical simulation calculation of the electromagnetic flanging forming process, the axial and radial displacement at the sheet metal hole edge basically no longer increases after 256 μs. Therefore, in this paper, only the influence of the 256 μs current at the first half wavefront on the electromagnetic flanging forming is considered. Subsequently, the influence of the sheet metal inertia effect of 244 μs on the flanging forming was conducted separately, and the entire cycle analysis time was 500 μs.

### 2.3. Finite Element Model of Structural Field

**Figure 4:**
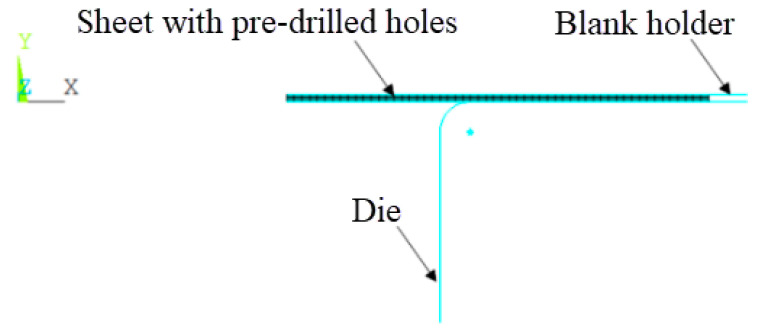
Structural field finite element model.

**Figure 5:**
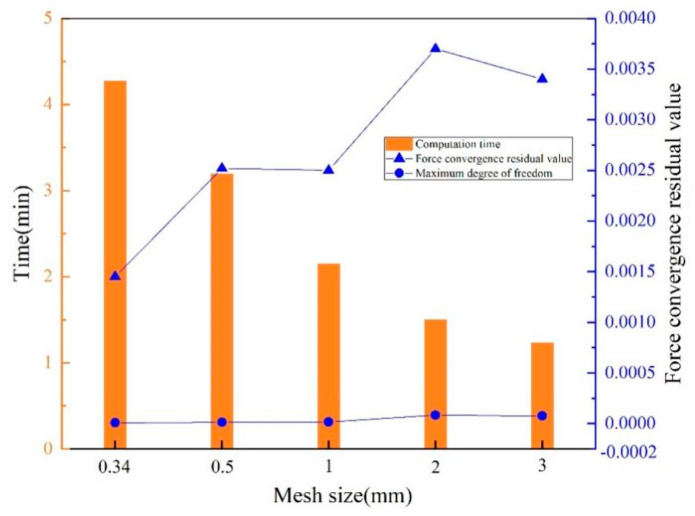
Analysis of grid rationality.

The field model of the circular hole electromagnetic flanging structure in the ANSYS APDL 2020 R2/ANSYS/MECHANICAL module is shown in Figure 4, which includes three parts: sheet metal, die and blank ring. Convert the sheet metal unit to the structural field unit Plane182, set the die and the blank holder as rigid bodies, set both the air and coil units to “null”, and set the flanging coefficient to 0.64. The magnetic field forces at each node on the sheet metal calculated by the ANSYS APDL 2020 R2//EMAG module are taken as the loads for structural analysis, and the elastoplastic deformation calculation of the sheet metal is carried out by introducing the self-compiled APDL program into the ANSYS APDL 2020 R2/MECHANICAL module. The grid rationality analysis is shown in Figure 5. The grid sizes analyzed are 0.34 mm, 0.5 mm, 1 mm, 2 mm, 3 mm. The selected analysis indicators are calculation time, force convergence value, and maximum degree of freedom. As shown in the bar chart in Figure 5, as the grid size increases, the calculation time gradually decreases. As indicated by the dotted line graph, the maximum degree of freedom changes slightly. When the grid size is 1 mm, the force convergence value is the second smallest among all. Considering all factors, the 1 mm grid size is selected for the calculation and analysis.

**Figure 6:**
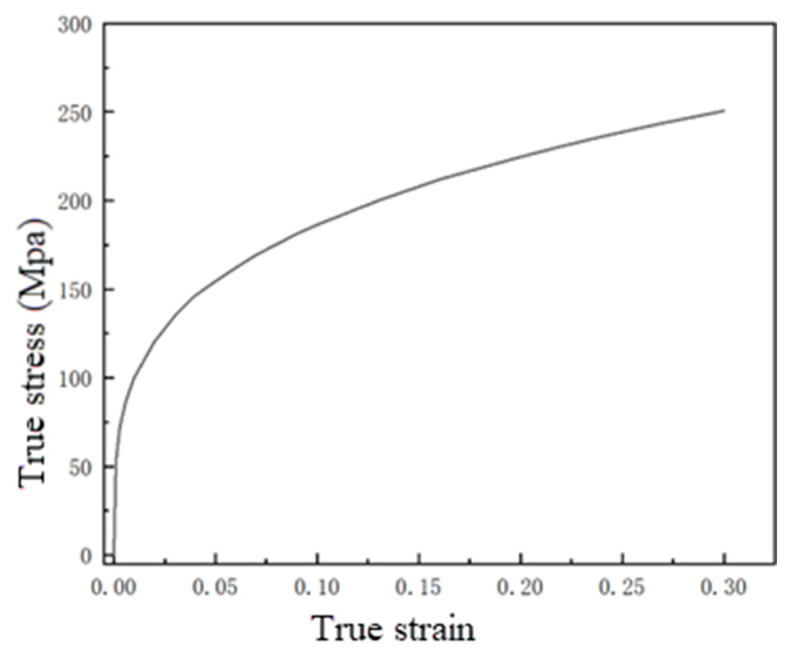
True stress–strain curve of aluminum alloy sheet (LF21M).

The workpiece material adopts annealed AA3003 (LF21M) aluminum alloy sheets, which were purchased from Shanghai Special Steel Co., Ltd., Dongguan, China, with a thickness of 1 mm, an inner diameter of 70 mm, and an outer diameter of 180 mm. The elastic modulus is 68.4 GPa, the Poisson’s ratio is 0.3, the yield strength is 74 MPa, and the tensile strength is 225 MPa. Figure 6 shows the true stress–strain curves of annealed AA3003 (LF21M) aluminum alloy sheet obtained from tensile tests at a speed of 3 mm/s using a unidirectional tensile testing machine at room temperature and quasi-static conditions [20].

Since the high strain rate in electromagnetic forming can change the constitutive relationship of the material, in this paper, the Cowper-Symonds constitutive model is adopted to take into account the influence of the material strain rate effect on the forming. The constitutive equation is shown as Equation (1):

(1) $$\sigma =\sigma_{y}\left(1+{\left(\frac{\dot{\varepsilon }}{p}\right)}^{m}\right)$$

In the formula, *σ* is the dynamic flow stress; *σ_y_* is the quasi-static flow stress (as shown in Figure 4); $\dot{\varepsilon }$ is the plastic strain rate/s^−1^; and *p* and *m* are strain rate constants. For aluminum alloys, *m* = 0.25 and *p* = 6500 s^−1^.

## 3. Finite Element Simulation Results

### 3.1. Distribution of Magnetic Field Lines

**Figure 7:**
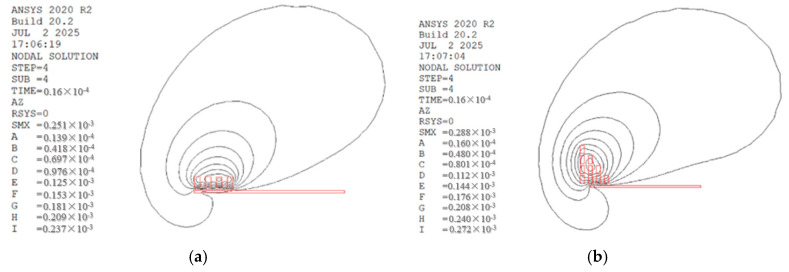
Distribution of magnetic field lines of electromagnetic flanging forming. (**a**) The flat coil and (**b**) the stepped coil.

Figure 7 shows the distribution of the magnetic field line density in electromagnetic flanging forming. The magnetic field lines are denser near the middle two turns of the flat coil than on the inner and outer sides, but there is a certain degree of concentration at the edge of the sheet hole because the inner diameter of the coil is flush with the edge of the sheet hole. Under the stepped coil, the magnetic field lines are the densest near the edge of the prefabricated hole, and gradually disperse along the radial direction outward. It can be analyzed from Figure 7. The magnetic field lines of the flat coil and stepped coil are represented by the letters A, B, C, D, E, F, G, H, I from the outside to the inside.

The magnetic field in the middle area of the flat coil is stronger than that on the inner and outer sides. Therefore, the electromagnetic force received by the sheet material corresponding to the middle area of the flat coil is greater than that on the inner and outer sides. The magnetic field intensity is the greatest at the edge of the pre-drilled hole, and therefore the electromagnetic force here is also the strongest at this area.

### 3.2. Distribution of Electromagnetic Force and Stress and Strain

At the initial moment of electromagnetic flanging forming, both the electromagnetic force and deformation received by the sheet metal are very small, and they increase rapidly with the deformation process. Moreover, there are significant differences in the electromagnetic force distribution generated by the two structural coils. To understand the influence of the electromagnetic force distribution of the two coils on the deformation of the sheet metal, a comparative analysis was conducted at a time of 64 μs, at which point the electromagnetic force begins to play a role in the forming of the sheet metal.

**Figure 8:**
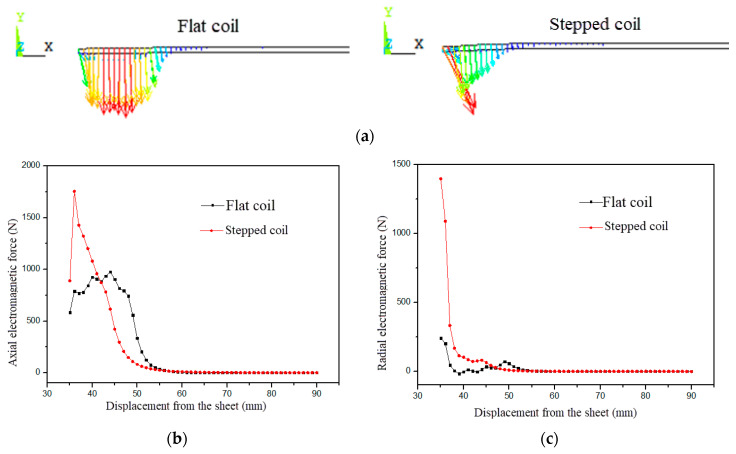
Distribution of electromagnetic force of sheets at 64 μs. (**a**) Electromagnetic force vector, (**b**) axial electromagnetic force and (**c**) radial electromagnetic force.

As shown in Figure 8, the electromagnetic force distribution of the sheet metal at 64 μs in electromagnetic forming is presented. It can be known from the vector distribution of the electromagnetic force of the sheet metal in Figure 8a that the electromagnetic force distribution of the flat coil is relatively large in the area of the sheet metal, and the electromagnetic force distribution in the middle of the sheet metal deformation zone is uniform with little difference. The electromagnetic force gradually and slowly decreases towards both sides of the middle of the deformation zone. The electromagnetic force distribution of the stepped coil is relatively narrow in the area of the sheet metal, mainly concentrated at the edge of the hole of the sheet metal, making the electromagnetic force at this location the greatest. The electromagnetic force gradually decreases along the radial direction of the sheet metal and outward. It can be known from Figure 8b,c that the axial electromagnetic force of the flat plate coil is evenly distributed on the sheet metal above the concave model cavity, and the axial electromagnetic force does not differ much. However, the axial electromagnetic force of the stepped coil is the greatest at the inner edge of the sheet metal and gradually decreases along the direction of the outer edge of the sheet metal. As can be seen from the electromagnetic force vector distribution diagram in Figure 8a, the radial electromagnetic forces of both the flat plate coil and the stepped coil are only distributed at the edge of the hole. Among them, the radial maximum electromagnetic force of the stepped coil is 1413 N, which is much greater than the radial electromagnetic force of the flat plate coil at the edge of the hole, and is about 5.6 times that of the flat plate coil.

**Figure 9:**
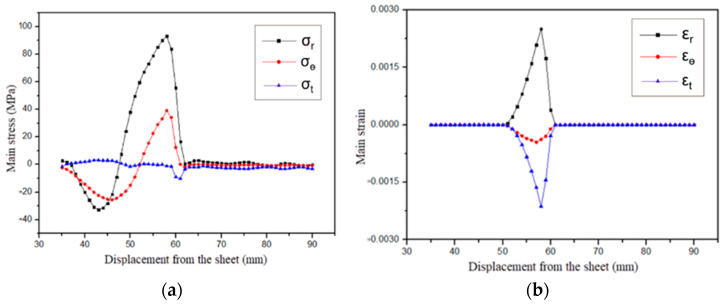
Distribution of principal stress and strain of sheets at 64 μs for flat coil. (**a**) Stress and (**b**) strain.

**Figure 10:**
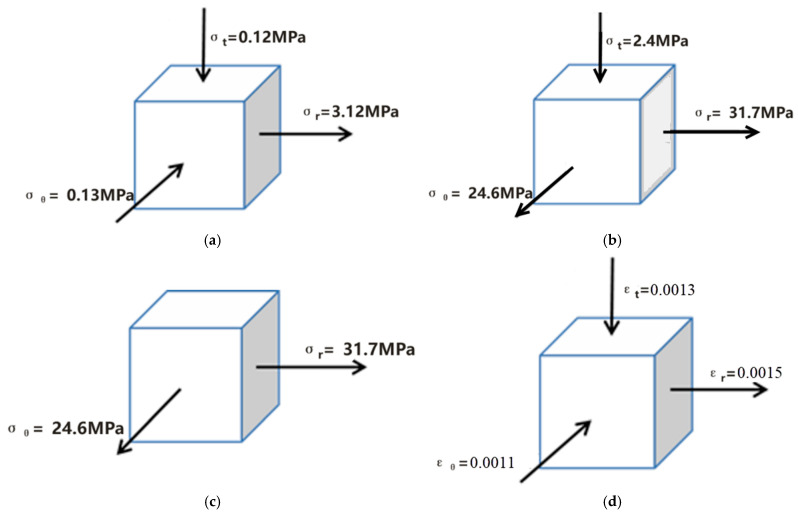
Diagram of principal stress and strain at the 64 μs moment. (**a**) Edge stress of the hole, (**b**) Midpoint stress, (**c**) Round corner stress and (**d**) Round corner strain.

Figure 9 shows the stress–strain distribution on the upper surface of the sheet metal at the 64 μs moment of the electromagnetic flanging forming of the flat coil. As shown in Figure 9a, from the edge of the inner hole along the radial direction outward, both the radial stress and the tangential stress change from compressive stress to tensile stress. Compared with the radial stress and the tangential stress, the axial stress is very small and does not change much. The electromagnetic force at the edge of the hole is relatively small, and at this time, it is not sufficient to drive the plastic deformation at the edge of the sheet metal hole. The sheet metal at the edge of the hole mainly moves under the drive of the middle of the deformation zone. The radial stress and tangential stress on the upper surface of the sheet metal in the edge area of the hole are compressive stress, and the axial stress is tensile stress. The principal stress diagram is shown in Figure 10a. Due to the increase in the density of magnetic field lines at the edge of the sheet metal hole and the center of the fillet, the electromagnetic force received increases, so the radial and tangential stresses at this location increase rapidly, as shown in Figure 10b. At the fillet, due to the reaction force exerted by the die on the sheet during deformation, both the radial and tangential stresses at this location change from compressive stress to tensile stress, as shown in Figure 10c. Strain mainly occurs at the fillet of the die, with tensile strain in the radial direction and compressive strain in the tangential and axial directions. The principal strain diagram is shown in Figure 10d. It can be known from the distribution of stress and strain in the sheet metal that the sheet metal at the fillet of the die undergoes plastic deformation first.

**Figure 11:**
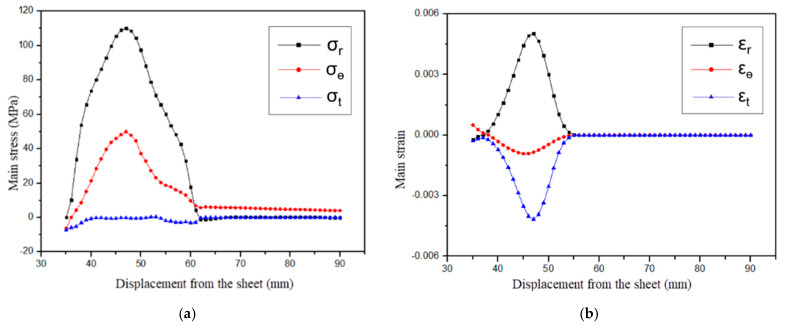
Distribution of principal stress and strain of sheets at 64 μs for stepped coil. (**a**) Stress; (**b**) strain.

**Figure 12:**
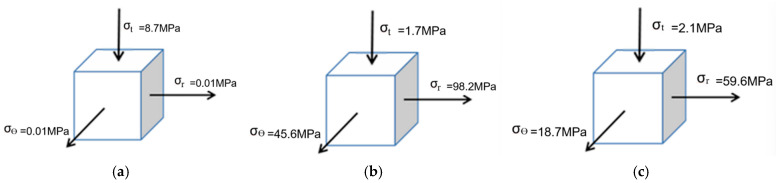
Main stress diagram, (**a**) hole edge stress, (**b**) mid-point stress and (**c**) rounded stress.

**Figure 13:**
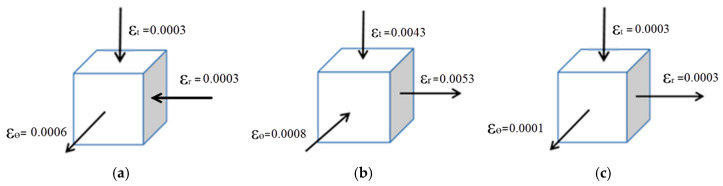
Diagram of principal strain, (**a**) hole edge strain, (**b**) mid-point strain and (**c**) rounded strain.

As shown in Figure 11, the stress–strain distribution on the upper surface of the sheet metal at 64 μs during the electromagnetic flanging forming of the stepped coil is presented. As shown in Figure 11a, the radial stress and tangential stress in the deformation zone of the sheet metal are both tensile stresses, and the stress in the thickness direction is compressive stress. Compared with the radial stress and tangential stress, it is very small. The principal stress diagram is shown in Figure 11. Since the axial and radial electromagnetic forces mainly act on the edge of the hole in the sheet metal, according to the yield criterion, it can be determined that the edge of the hole is the first part to undergo plastic deformation, and the thickness reduction is also the most severe. The strain of the sheet metal gradually decreases from the edge of the hole to the fillet of the die. Compared with the flat plate coil, the step coil has tangential tensile stress at the edge of the hole, which is conducive to the forming of the sheet metal at the edge of the hole. This makes the radial and tangential stresses of the sheet metal in the entire deformation zone tensile stresses, which is manifested as the elongation of the sheet metal as a whole. Figure 11b shows the strain distribution at different positions. At the hole edge, the radial and tangential directions represent tensile strain, and the axial direction represents compressive strain. The principal stress diagram is shown in Figure 12a. The points on the stress diagram at the edge of the hole and the rounded corners, as well as the principal stress diagram at the rounded corners, are shown in Figure 12b,c.The principal strain diagram is shown in Figure 13a. The electromagnetic force applied by the stepped coil on the sheet metal gradually decreases from the edge of the hole to the fillet. The principal strain diagrams at the midpoints of the edge of the hole and the fillet and at the fillet are shown in Figure 13b,c. The magnetic field line distributions of both types of coils extend from the edge of the hole to the fillet of the die. However, the maximum density of the magnetic field lines of the flat coil is distributed in the middle of the deformation zone of the sheet material, while that of the stepped coil is distributed at the edge of the hole. Moreover, the tangential tensile stress at the edge of the hole is much greater than that of the flat coil. In the early stage of forming, the electromagnetic force required for deformation at the edge of the hole is the greatest. The magnetic field line distribution mode of the stepped coil can further increase the flanging height compared with the flat coil and effectively reduce the problem affecting the quality of the flanging part due to the large gap of the right-angle edge.

Circular hole flanging belongs to the elongation type of flanging [21,22]. During flanging, the tangential tensile stress σ_θ_ in the deformation zone generates tangential tensile strain ε_θ_, and the effect is manifested as the tangential elongation of the sheet metal and thinning of the sheet metal thickness. The radial strain ε_r_ generated by the radial tensile stress σ_r_ in the deformation zone during flanging is relatively small. Compared with the flat coil, the step coil can apply a greater force to the sheet metal during the flanging process. Sufficient forming pressure is the key to ensuring the success of flanging. Meanwhile, the flanging speed of the sheet metal in the step coil experiment is more reasonable, avoiding the problem of sheet metal springback caused by too small a flanging speed due to less pressure [23,24]. To sum up, during the flanging process, the deformation zone near the hole edge is mainly subjected to a bidirectional tensile stress state (the stress in the thickness direction of the plate is ignored). The radial compressive stress and tangential compressive stress generated at the edge of the hole during the forming process of the flat coil are not helpful for the flanging deformation. The radial tensile stress and tangential tensile stress generated at the hole edge during the forming process of the stepped coil are conducive to the flanging deformation. Moreover, according to the yield criterion, it can be determined that the edge of the hole is the part where plastic deformation occurs first, and the thickness reduction is also the most severe. The strain of the sheet metal gradually decreases from the edge of the hole towards the fillet of the die. The plastic deformation of the step coil mainly occurs at the edge of the hole, which is conducive to flanging forming.

**Figure 14:**
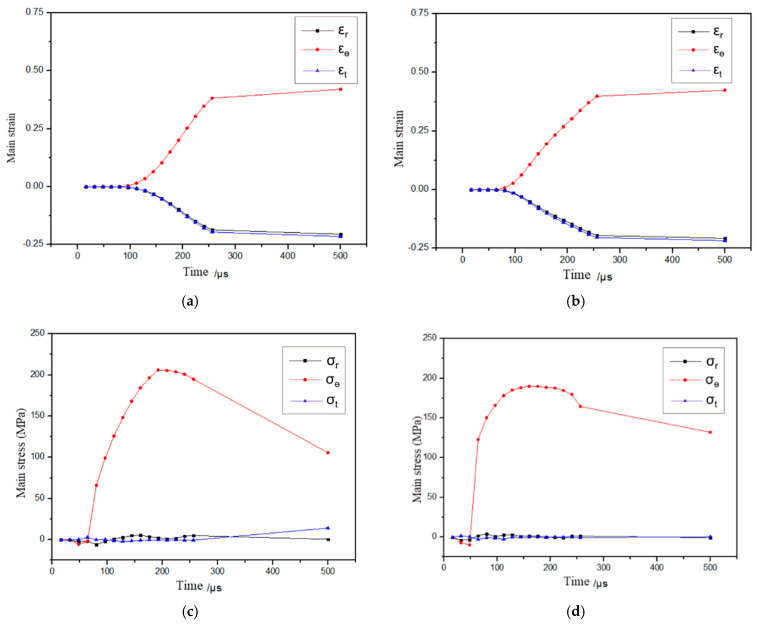
Variation curve of stress and strain with time at hole edge of the sheet. (**a**) Strain (the flat coil), (**b**) strain (the stepped coil), (**c**) stress (the flat coil) and (**d**) stress (the stepped coil).

In the flanging forming process, the inner edge of the sheet metal is an area prone to cracking defects [25,26]. The stress and strain at the edge of the sheet metal hole should be analyzed. The relationship between the stress and strain at the edge of the sheet metal hole and time is shown in Figure 14. As shown in Figure 14a,b, the strain variations indicate that during the electromagnetic flanging forming of the flat plate coil and the step coil, the hole edge of the sheet is in a state of bidirectional radial and thickness compressive strain and tangential tensile strain. As shown in Figure 14c,d, during the electromagnetic flanging forming of the flat plate coil and the step coil, the inner hole edge of the sheet is mainly subjected to tangential tensile stress. It can be known from the electromagnetic force distribution of the stepped coil and the flat coil that the electromagnetic force received at the edge of the hole of the stepped coil at the initial moment is greater than that of the flat coil, and the stress at the edge of the hole increases faster.

As shown in Figure 14b,c, the maximum tangential tensile stress of the stepped coil is 196.48 MPa, which is 7.75% smaller than that of the flat coil. The reduction in the tangential tensile stress effectively reduces the thinning of the plate in the tangential direction. Therefore, the electromagnetic flanging forming of the stepped coil is more conducive to preventing the occurrence of tensile cracking defects. During the inertial forming period of 256–500 μs, the decrease in tangential tensile stress at the inner hole edge of the stepped coil during forming is smaller than that of the flat coil. This indicates that when the inner hole edge of the sheet metal collides with the die during the forming of the stepped coil, the reaction force it receives is smaller than that of the flat coil, which is more conducive to the die adhesion between the hole edge and the inner wall of the die.

For traditional flanging forming, the inner hole flanging is mainly carried out by reducing the thickness of the sheet metal at the edge of the hole. Uneven thickness reduction from the fillet of the sheet metal die to the edge of the hole is prone to cracking [27]. The edge of the hole is only subjected to tangential tensile stress. In electromagnetic flanging, in addition to tangential tensile stress, it is also subjected to radial tensile stress, but the radial tensile stress is less than the tangential tensile stress, so it is manifested as tangential tensile strain. The radial and axial directions are compressive strain, and the axial compressive strain is greater than the radial compressive strain. Therefore, compared with punch flanging, electromagnetic flanging increases the electromagnetic force that promotes radial tensile. The thickness thinning near the edge of the electromagnetic flanging hole is more severe, and at the same time, the flanging height is also higher.

### 3.3. Deformation Analysis of Sheet Metal at Different Moments

**Figure 15:**
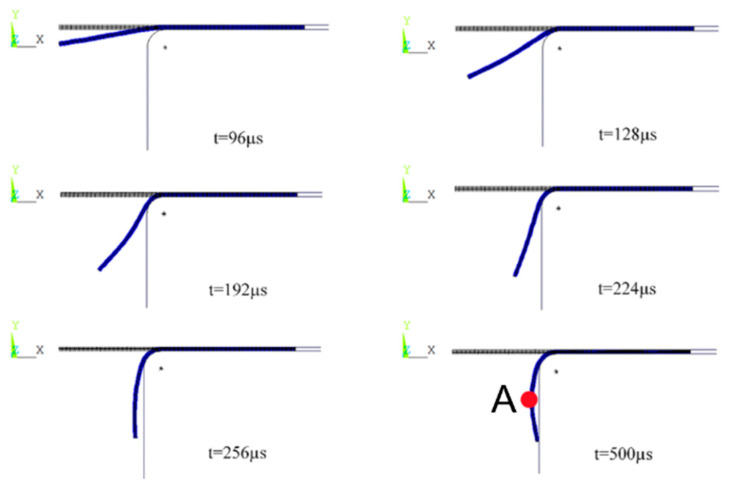
Structural deformation diagram of sheets at different times for flat coil.

Figure 15 shows the structural deformation law of the sheet metal formed by the electromagnetic flanging of the flat coil at different times. It can be seen that the sheet metal mainly undergoes plastic deformation between 96 μs and 256 μs. It can be known from Figure 7 that in the early stage of forming, the middle part of the sheet metal deformation zone deforms first due to the drive of electromagnetic force. The sheet metal at the edge of the hole undergoes plastic deformation under the drive of the sheet metal in the middle of the deformation zone with the effect of inertia. In the later stage of forming, compared with the sheet metal near the middle of the deformation zone, the sheet metal at the edge of the hole shows a “leading” phenomenon under the effect of inertia. In the later stage of forming, although the current density continues to increase, the distance between the sheet metal in the middle of the deformation zone and the coil also increases. At this time, the magnetic flux passing through the sheet metal decreases, and the magnetic flux generated by the induced current will be hindered and decrease [28,29]. Therefore, the forming speed in the middle of the deformation zone that deforms first slows down, while the sheet metal at the edge of the hole (which has a greater speed due to inertia) comes into contact with the inner wall of the die first and collides, causing the sheet metal near the middle of the deformation zone (point A) to fail to adhere to the die. Eventually, this results in the largest gap between the middle of the sheet metal deformation zone and the inner wall of the die, and the flanging quality is poor.

**Figure 16:**
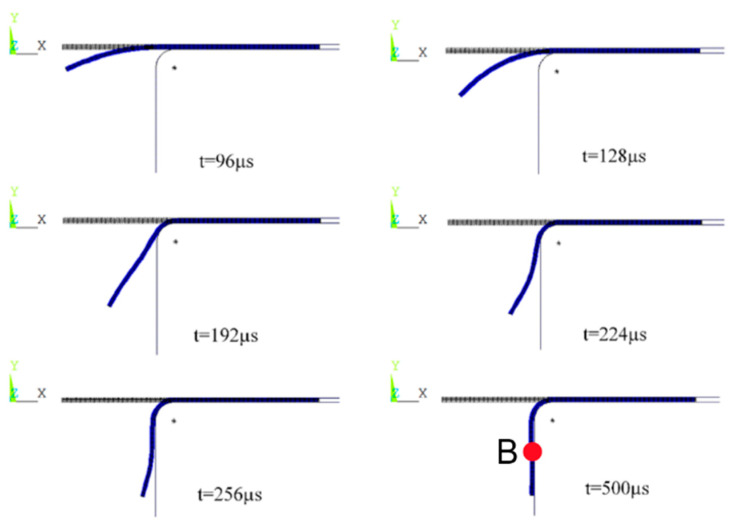
Structural deformation diagram of sheets at different times for gradient coil.

Figure 16 shows the structural deformation law of the sheet metal formed by the electromagnetic flanging of the step coil at different times. During the electromagnetic flanging forming process of the step coil, the inner edge of the hole of the sheet metal undergoes plastic deformation first compared to other areas of the sheet metal in the early stage of forming, and the sheet metal presents a convex shape. And as can be seen from Figure 8, since the electromagnetic force is mainly concentrated at the edge of the hole in the early stage of forming, under the driving effect of the electromagnetic force, the sheet metal at the edge of the hole undergoes plastic deformation first. As the forming process proceeds, the electromagnetic force gradually shifts to the middle of the deformation zone. The electromagnetic force acting on the middle part of the sheet material gradually increases. Driven by the sheet material at the edge of the hole, the middle part of the deformation zone of the sheet material (point B) first comes into contact with the inner wall of the die. The edge of the hole of the sheet material mainly relies on the effect of inertia to continue deforming and successively comes into contact with the inner wall of the die. Finally, the minimum film distance of the sheet material is 0.05 mm. The film application effect is very good and the flanging quality is very high.

From the above analysis, it can be known that the stepped coil electromagnetic flanging can make the deformation zone of the sheet metal flanged into a vertical edge, and the flanging quality is better than that of the flat coil electromagnetic flanging, with a smaller die gap. Therefore, the spatio-temporal distribution law of the electromagnetic force acting on the sheet metal during the electromagnetic flanging forming process of two different structural coils can be further analyzed to reveal the superiority of the stepped coil electromagnetic flanging forming process.

### 3.4. The Influence of Sheet Metal Electromagnetic Force Distribution on Deformation

**Figure 17:**
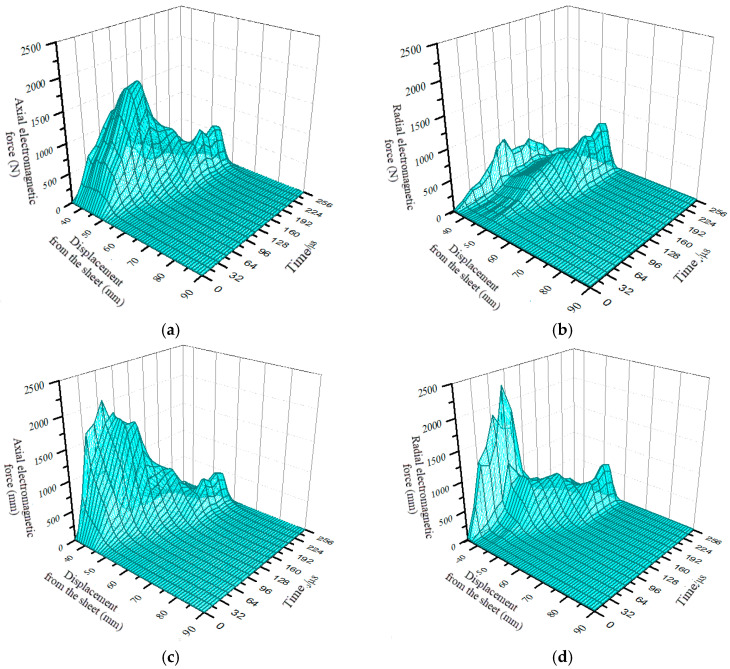
Curve of electromagnetic force distribution over time in different positions of sheet material. (**a**) Axial (the flat coil), (**b**) radial (the flat coil), (**c**) axial (the stepped coil) and (**d**) radial (the stepped coil).

The axial electromagnetic forces at different positions on the sheet metal are obtained by superimposing the axial electromagnetic forces at the same radial position node of the sheet metal in the electromagnetic field. The radial electromagnetic force is obtained by superimposing the radial electromagnetic forces at the same radial position node of the sheet metal in the electromagnetic field. The distribution of the electromagnetic force at different positions of the electromagnetic flanging forming sheet metal over time is shown in Figure 17.

In the early stage of forming, the axial electromagnetic force of the flat plate coil is evenly distributed in the sheet metal deformation area above the concave mold cavity. The radial electromagnetic force is concentrated at the inner edge of the sheet metal, but it is not much different from that in other areas of the sheet metal. The axial electromagnetic force of the stepped coil is mainly concentrated at the hole edge of the sheet metal. Along the radial direction outward, the electromagnetic force gradually decreases. The axial electromagnetic force at the hole edge of the sheet metal reaches the maximum value of 2058.7 N at 80 μs. The radial electromagnetic force is mainly concentrated at the hole edge of the sheet metal, which differs greatly from that in other areas of the sheet metal, reaching the maximum value of 2284.3 N at 96 μs. In the middle stage of forming, as the deformation of the sheet metal keeps increasing and the distance from the coil keeps increasing, both the axial and radial electromagnetic forces gradually shift along the diameter to the middle of the deformation zone of the sheet metal. The axial electromagnetic force of the flat plate coil reaches its maximum value of 1774.3 N at 112 μs and a radial distance of 45 mm. Compared with the electromagnetic flanging of the step coil, the axial electromagnetic force response of the sheet material is slower. In the later stage of forming, the distance between the sheet metal and the coil at the fillet of the die is the closest. The electromagnetic forces are all concentrated on the sheet metal in this area. Therefore, the axial and radial electromagnetic forces of the flat plate coil and the step coil are all concentrated on the sheet metal at the fillet of the die and reach the maximum value.

To sum up, as mentioned above, the distribution area of the electromagnetic force at different positions of the sheet metal formed by electromagnetic flanging over time is relatively narrow, and both the axial and radial electromagnetic forces are mainly distributed near the edge of the sheet metal hole. The electromagnetic force distribution area of the sheet metal in the electromagnetic flanging of the flat plate coil is relatively wide. The axial electromagnetic force is mainly distributed in the middle of the sheet metal deformation zone, and the radial electromagnetic force is distributed throughout the entire deformation zone of the sheet metal. The maximum radial electromagnetic force of the sheet metal in the electromagnetic flanging of the stepped coil is much greater than that of the sheet metal in the electromagnetic flanging of the flat coil. Moreover, the maximum axial electromagnetic force of the electromagnetic flanging of the stepped coil occurs at the hole edge of the sheet metal in the middle of the forming process. In the middle of the forming process, the radial electromagnetic force is also uniformly distributed in the middle of the sheet metal deformation zone. The maximum axial electromagnetic force of the electromagnetic flanging of the flat coil occurs at the fillet of the die in the later stage of forming for the sheet metal. Therefore, the plastic deformation of the stepped coil electromagnetic flanged sheet material occurs under the dual drive of axial electromagnetic force and radial electromagnetic force, while the flat coil electromagnetic flanged sheet material mainly undergoes plastic deformation under the drive of axial electromagnetic force.

### 3.5. Energy Distribution of Sheet Metal at Different Time

**Figure 18:**
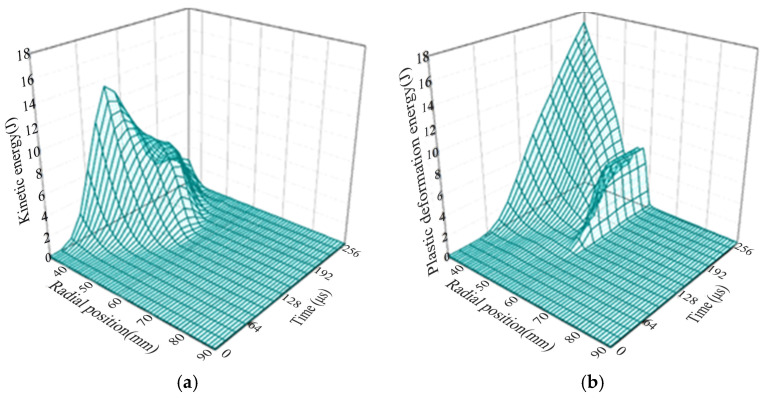
Curve of energy distribution with time at different positions of sheet metal. (**a**) Sheet metal kinetic energy and (**b**) plastic deformation energy of sheet metal.

Since the energy variation trend of the flat plate coil electromagnetic deep drawing forming over time is basically the same as that of the step coil, only the energy variation relationship of the step coil electromagnetic deep drawing forming over time is further analyzed here. As shown in Figure 18, the distribution curves of the kinetic energy and plastic deformation energy of the electromagnetic flanged sheet material of the step coil at different positions over time are presented. As shown in Figure 18a, the distribution law of the kinetic energy of each part of the sheet material is related to the magnetic field and magnetic force induced by the coil on the sheet material. In the early stage of forming, the kinetic energy at the edge of the hole is relatively large, and in the middle stage of forming, the kinetic energy gradually transfers to the middle of the deformation zone. Figure 18b shows the variation in the plastic deformation energy of the sheet metal. The plastic deformation energy of the sheet metal at the edge of the hole is large, and the plastic deformation energy mainly occurs within the range of 96–256 μs. From the perspective of energy, the electromagnetic force concentration degree of the stepped coil at the edge of the sheet metal hole is higher, and the required plastic deformation energy here is also higher. Therefore, compared with the flat coil, the structure of the stepped flanged coil is more reasonable.

## 4. Experimental Verification of Electromagnetic Flanging Forming Process

### 4.1. Electromagnetic Flanging Process Test Tooling

**Figure 19:**
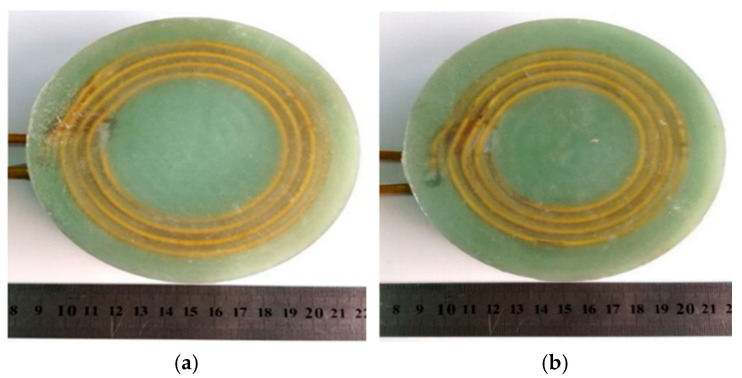
Two different structural coils. (**a**) The flat coil; (**b**) the stepped coil.

**Figure 20:**
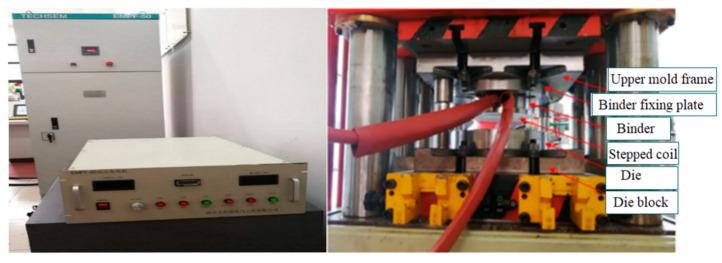
Electromagnetic forming machine EMPF-50 and Electromagnetic flange experiment fixture.

The flat coil and step coil are shown in Figure 19. Figure 20 shows the electromagnetic forming machine and the test fixture for electromagnetic bending forming.

**Figure 21:**
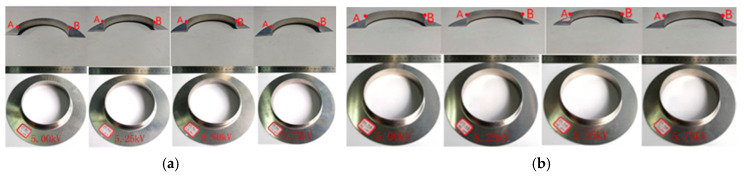
Forming results of the flanged parts under different voltage conditions. (**a**) The flat coil; (**b**) the stepped coil.

The cross-sectional shape of the AA3003 aluminum alloy formed by the electromagnetic flanging of a flat coil is shown in Figure 21a, while that formed by the electromagnetic flanging of a stepped coil is shown in Figure 21b, points A and B in the figure represent the edge positions of the flange. The edge-pressing force is calculated as 36 kN per unit edge-pressing force of 2.5 MPa [14], and the discharge voltages are taken as 5 kV, 5.25 kV, 5.50 kV, and 5.75 kV, respectively. Under the action of voltages of 5 kV, 5.25 kV, 5.50 kV and 5.75 kV for the flat coil, the thicknesses of the flanged parts at points A and B are consistent, which are 0.7 mm, 0.7 mm, 0.6 mm and 0.5 mm, respectively. The flanged height is 22.15 mm. The flanged parts are all curved edges with a certain arc and have a large gap with the inner wall of the die. It cannot be turned into a vertical edge. Under the action of voltages of 5 kV, 5.25 kV, 5.50 kV and 5.75 kV for the stepped coil, the thicknesses of the flanged parts at points A and B are the same, both 0.6 mm, and the flanging height is 23.17 mm. The sheet materials can eventually be flanged into vertical edges. The flanging height is higher than that of the flat coil, and the flanging quality is better. This is consistent with the result of the deformation of the sheet metal structure obtained through simulation.

### 4.2. Comparison of the Diameter of the Middle Part of the Deformation Zone of the Flanging Piece

**Figure 22:**
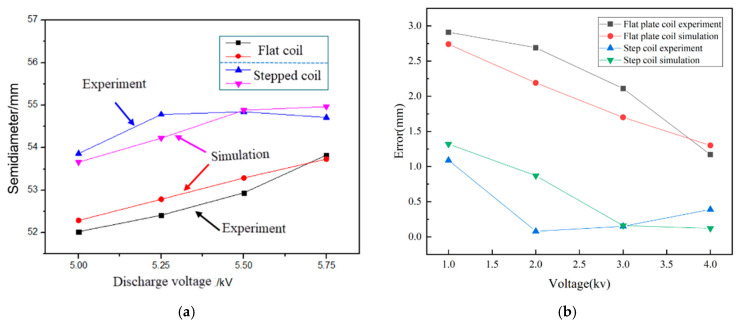
(**a**) Comparison of diameter in the middle of the deformed workpieces and (**b**) experimental and simulation error of flat and stepped coil.

The final flanging radius of the middle part of the deformation zone of the flanged part formed under the conditions of 5 kV, 5.25 kV, 5.50 kV, and 5.75 kV (i.e., points A and B at the initial radius of 45 mm, as shown in Figure 15 and Figure 16, is shown in Figure 22a. Under these four different voltage conditions, the radius of the middle deformation zone of the stepped coil during flanging is larger than that of the flat coil, which allows for better adherence to the mold. In the electromagnetic flanging forming experiment, the maximum flanging radius in the middle of the deformation zone for the flat coil was 53.83 mm at 5.75 kV, while for the stepped coil, the maximum flanging radius in the middle of the deformation zone was 54.85 mm at 5.5 kV. Moreover, the simulation results are close to the experimental results, and the maximum relative error is 1.01%; the specific errors are shown in Figure 22b. The maximum and minimum errors of the stepped coil electromagnetic flanging experiments are 1.12 mm and 0.11 mm, respectively, which are approximately 40% and 9% of the maximum and minimum values of the flat coil experiments.

## 5. Conclusions

(1) This paper proposes a stepped coil electromagnetic flanging forming process and conducts finite element numerical simulation. Combined with the experimental results, it shows that the stepped coil electromagnetic flanging can turn the sheet metal into a vertical edge with good flanging quality, verifying the feasibility of the stepped coil electromagnetic flanging forming process.
(2) In the initial stage of formation, the electromagnetic forces exerted by the two different structures of coils on the sheet material were different (the maximum electromagnetic force of the stepped coil was distributed at the edge of the hole, with a value of 1413 N, while the maximum electromagnetic force of the flat coil was located at the projection point of the third-turn coil on the sheet material, with a value of 253 N), resulting in different positions for the generation of stress and strain, and different positions where the sheet material began to undergo plastic deformation.
(3) Compared with the electromagnetic flanging of flat coil, in the forming process of stepped coil electromagnetic flanging, the radial electromagnetic force also has an effect on the plastic deformation of the sheet metal. Compared with the flat coil, the electromagnetic flanging of the stepped coil more effectively suppresses the occurrence of defects such as sheet material cracking at the hole edge, and is more conducive to the sheet material adhering to the mold at the hole edge.
(4) In the electromagnetic flanging forming experiment, the diameter of the middle part of the deformation zone of the flanging parts obtained by the step coil was larger than that of the flat coil, which could better adhere to the mold and had a high forming accuracy, and the simulation results were close to the experimental results, with the maximum relative error being 1.01%. The flanging diameters obtained from both the stepped coil experiment and the simulation both increased with the increase in voltage. Among them, the maximum flanging diameter obtained from the experiment was 109.78 mm, while the maximum flanging diameter obtained from the simulation was 109.84 mm.

## Figures and Tables

**Table 1:** Electromagnetic field model material parameters.

Material	Property	Value
Far field air	Relative permeability	1
Air	Relative permeability	1
Coil	Relative permeability	0.999994
Sheet	Resistivity ρ/Ω.m	1.72 × 10^−8^
	Relative permeability	1.0002
	Resistivity ρ/Ω.m	3.4 × 10^−8^

## Data Availability

The original contributions presented in this study are included in the article. Further inquiries can be directed to the corresponding author.
